# PLANETARY GRAND CHALLENGES: A CALL FOR INTERDISCIPLINARY PARTNERSHIPS

**DOI:** 10.24926/ijps.v6i1.1976

**Published:** 2019-04-11

**Authors:** Marie Gilbertson, Meggan Craft, Teddie Potter

**Affiliations:** College of Veterinary Medicine at the University of Minnesota; College of Veterinary Medicine at the University of Minnesota; University of Minnesota School of Nursing

**Keywords:** Climate change, planetary health, rabies, Science of Team Science, ecosystem services, climate curriculum, effective communication, domination paradigm, leadership, transdisciplinary team

## Abstract

Universities have traditionally been places where individual scholars work on individual topics, in individual disciplines, with individual funding. Even though large research institutions include all the major disciplines, faculty and students remain in their schools or colleges, rarely crossing the campus to interact. Matters do not improve once knowledge is generated. Each discipline has its own journals, its own conferences, and its own professional organizations. The academy was designed to support unparalleled expertise in specialized knowledge. However, universities are beginning to realize that the greatest challenges we face are systems problems and can only be solved by systems thinking and systems solutions. Climate change, antibiotic resistance, water scarcity, and unsustainable population growth are just a few of the planetary health crises that require interdisciplinary partnerships to solve. Fortunately, we are beginning to see early signs of a shift toward, and even normalization of, interdisciplinary collaboration. In fact, some national grants require team members from different fields as a stipulation for funding. Interdisciplinary research permits cross-field benefits in which the synergy of two or more knowledge sets is greater than the sum of its parts. Innovation increases and previously elusive solutions become possible. The field of partnership studies closely aligns with the vision and mission of interdisciplinarity and offers a philosophical framework to guide teaching and research.

## PLANETARY HEALTH

Publication of the Rockefeller Foundation-Lancet Commission report, *Safeguarding Human Health in the Anthropocene Epoch* (2015), initiated a shift from previously siloed approaches to improving human health to a systems approach known as planetary health. Planetary health considers the health of human civilization to be interconnected with the health of the entire planetary ecosystem. If the planetary ecosystem is threatened and not sustainable, human existence is no longer sustainable. The two are inseparable because they are one.

The shift to interdisciplinary thinking does not happen automatically. The academy was founded on the fragmentation of knowledge. Most faculty members are products of fragmented education, and despite “flipped” classrooms and active learning environments, the old “sage on the stage” model of teaching often persists. Planetary health requires thinking differently, learning differently, and engaging across disciplines in new ways.

## PARTNERSHIP STUDIES

Riane Eisler’s (1988) cultural transformation theory [CTT] offers a theoretical framework to support the transition from fragmented thinking to systems thinking, and from human domination to planetary interconnection. According to CTT, all human social networks, from families to organizations to nations, are structured on a continuum that ranges from domination to partnership.

The domination and partnership paradigms have classic characteristics. Social structures that orient toward domination are rigidly ranked, and communication only flows one way. The leader uses “power over” approaches to maintain the current power structure, and shame, blame, and fear are classic techniques for control and hierarchical stability. If a university orients toward domination, the “hard sciences” are often valued above art and literature; even within the sciences, one school or college, such as medicine, may be deemed more important than the other health professions.

In these universities, domination is the pattern in classrooms as well. The faculty determines the topics, the content that will be taught, the texts and literature that will be required reading, and the standards for success. In extreme domination systems, students fear reprisals if they voice opinions that differ from the faculty or if they challenge professors who claim the students’ work as their own.

Domination cultures value men over women, support marginalization of certain races and identities, and promote economic wealth over human health and immediate gratification over the needs of future generations. Perhaps one of the most egregious forms of domination is the rigid hierarchy that encourages humans to dominate nature.

Partnership systems on the other hand have completely different structural characteristics. Partnerships are notable for their high level of mutual respect. Each member is highly valued, and unique contributions are recognized and appreciated. Communication flows both ways, and risk-taking and innovation are encouraged. There are still leaders in partnership systems, but they use “power with” approaches to lift everyone to reach their full potential.

If a university orients toward partnership, all disciplines are valued and respected for the way they contribute to the whole. If one college or school struggles, the other schools are supportive and do what they can to lift the other up.

In the classroom, partnership faculty understand that students have ideas to share, experiences to tap, and learning needs that may differ from the curriculum designed by the faculty. Faculty understand that in the future, students will work and lead in contexts that may be very different from the systems of today, and need to learn new content, gain emerging skills, and work across sectors to solve complex problems.

In partnership-based health care, one profession does not dominate another. In fact, each has a clear grasp of the contributions of the other professions. When an issue arises that falls within the expertise of one particular profession, the others work in partnership but defer leadership to the profession with the greatest expertise. If your house is burning down, firefighters are called to handle the situation and lead the response. It is neither safe nor practical for any others to lead the effort to extinguish the flames.

## PARTNERSHIP-BASED PLANETARY HEALTH

Planetary health can also be approached from a domination or partnership perspective, and the nature of the perspective plays a significant role. A domination approach to planetary health involves “parallel play.” Even though the complexity of the issue is recognized, and it is understood that multiple specialties need to be part of the solution, the specialties may work separately and engage each other infrequently. When they do engage, there is a tendency to rank the ideas or contributions of one discipline above another. In addition, the needs and wants of humans are ranked above the health of the entire ecosystem. Approaching planetary health from a partnership perspective encourages equity among the members of an ecosystem. Value is placed on the health of the whole rather than defaulting to the needs and wants of humans. Future generations also have a shared stake and value. Deep humility and respect are hallmarks of a partnership approach to planetary health. It is understood that effective and sustainable solutions only come when the needs of the whole are considered.

### Why is partnership a critical approach to complex problems? Lessons from the natural sciences

Interdisciplinary partnerships have been increasingly called upon to tackle complex, messy planetary grand challenges, including those relating to health. The need for partnerships is apparent when considering zoonotic diseases that are shared between humans and animals. Many emerging infectious diseases spill over from animals, create a media frenzy with their pandemic potential, and often garner access to global investment in resources (e.g. Nipah virus, severe acute respiratory syndrome [SARS], pandemic influenza, Ebola virus, Zika virus, and Middle East respiratory syndrome [MERS]). In contrast to emerging infectious diseases, endemic zoonoses (e.g. trypanosomiasis, leptospirosis, brucellosis, and rabies) cause a quiet, persistent loss of lives and disability-adjusted life years. Finding causes and solutions to zoonoses, particularly in developing nations, has been limited by “disciplinary specialisms, sectoral mandates, divided policy efforts, and compartmentalized funding flows” (Cunningham, Scoones, & Wood, 2017, p. 1). Because zoonoses are caused by pathogens with complex life cycles that involve multiple host species and vectors, the burden of zoonoses cannot be reduced by utilizing expertise only from the medical silo, where treatment is focused solely on human cases. For example, a focus on treating humans for zoonoses such as brucellosis, leptospirosis, or Q fever in poor areas in Tanzania misses the root cause of the problem: that animals are maintaining the transmission cycle of the pathogens (Cleaveland et al., 2017). The most efficient and cost-effective way of reducing, and possibly eliminating, zoonoses involves reducing incidence in the animal host and/or reducing risky animal-human contacts (Cleaveland et al., 2017).

The contrast between tackling complex health challenges from a disciplinary or a partnership perspective is well illustrated by rabies. Canine rabies is a multi-host zoonotic disease transmitted by biting. It can infect all species of mammals, and can kill wildlife, domestic animals, and humans. Rabies is maintained by unvaccinated domestic dog and/or wildlife populations, while humans are ‘dead end’ hosts (Nel et al., 2005). If prevention and treatment of rabies was addressed by one disciplinary silo (i.e., human health), then humans who are in close contact with potentially rabid animals would be vaccinated, other humans would be advised to stay away from unvaccinated domestic dogs and wildlife, and post-exposure prophylaxis would be given to those citizens who could access and afford it (Hampson et al., 2015; Knobel et al., 2005). Rabies would continue to persist if only the dead-end hosts were considered.

Alternatively, a partnership approach looks at the rabies system holistically in order to identify the species or set of species that is maintaining the rabies infection in the ecosystem (Haydon, Cleaveland, Taylor, & Laurenson, 2002; Lembo et al., 2008). If the maintenance population involves domestic dogs, domestic dogs would be vaccinated (Cleaveland et al., 2018); if the maintenance population involves wild animals, they could be vaccinated with oral baits (Elmore et al., 2017; Müller & Freuling, 2018). Targeting the maintenance population could ultimately eliminate rabies in a system and prevent the loss of human lives across all socio-economic classes (Cleaveland & Hampson, 2017).

A partnership and planetary health approach to rabies control appears to be working in Tanzania. Here, local veterinary and medical officers around the Serengeti ecosystem partnered with foreign researchers to employ annual domestic dog vaccination in rural communities (Hampson et al., 2009). As losses in human and animal lives decreased, mass dog vaccination was shown to be feasible, and doubts about elimination of canine rabies were dispelled (Kaare et al., 2009; Lembo et al., 2010). This domestic dog vaccination program gained popularity in both the medical and veterinary sectors, and grew in scope (Cleaveland & Hampson, 2017; Cleaveland et al., 2018). With this robust recent research on the feasibility of eliminating canine rabies through mass dog vaccination, in 2012 rabies was added to the World Health Organization’s (WHO) Accelerated Roadmap for Neglected Tropical Diseases (WHO, 2012). Then in an exemplary partnership between the human health and animal health sectors in 2015, the WHO and the World Organisation for Animal Health (OIE) set a goal to eliminate dog-mediated human rabies globally by 2030 (World Health Organization, Food and Agriculture Organization of the United Nations, & World Organisation for Animal Health, 2016).

### Promoting stakeholder buy-in

Stakeholder support is vital to the success of partnerships addressing complex health problems. The buy-in of medical and veterinary professionals - and eventually policymakers - promoted sustainability and collective action in addressing the rabies challenge. Achieving this kind of stakeholder buy-in, however, is a challenge for interdisciplinary partnerships, as various stakeholders are often driven by different priorities and values. Clarifying and speaking to the values of planetary health partners is therefore necessary, and identifying points of common language may support communication across stakeholders.

A common language strategy might include concepts such as ecosystem services. The ecosystem services framework emphasizes characterizing and quantifying the benefits humans derive from healthy ecosystems (Costanza et al., 1997; Travis et al., 2018). A classic ecosystem services success story is the protection of New York City’s watershed. Preserving and restoring the watershed provided New York City with clean drinking water at a fraction of the cost of building an engineered filtration plant (Chivian & Bernstein, 2008; Postel & Thompson, 2005). Here, ecosystem services defined the economic value of conserving a healthy ecosystem, thereby speaking to the values of both conservationists and policy makers. By providing a common language in line with the goals and vision of planetary health, ecosystem services can be an effective tool for communication in partnership approaches.

Ecosystem services can also help to describe the link between healthy ecosystems and healthy humans and animals (Bayles et al., 2016; Travis et al., 2018). In this sense, “health” becomes a common language which links the values of stakeholders. For example, climate change is causing shifts in range and prevalence of vector-borne diseases (e.g. Lyme disease and West Nile virus) on a global scale (Beard et al., 2016; Medlock & Leach, 2015; Ryan, Carlson, Mordecai, & Johnson, 2019). Highlighting such tangible consequences of climate change provides a connection between the values of climate activists, health professionals, policy makers, and the general public who are directly experiencing these health effects. Further, the health of domestic animals and wildlife are also affected by these climate-driven changes to vector-borne disease ecology (Altizer, Ostfeld, Johnson, Kutz, & Harvell, 2013; Gale, Drew, Phipps, David, & Wooldridge, 2009), making health a uniting value and common language across stakeholders.

Acknowledging and speaking to the different value systems of partners and stakeholders is necessary for tackling complex planetary grand challenges, and may necessitate dynamic communication strategies when addressing diverse priorities. This flexibility could be thought of as “code switching,” in which interdisciplinary teams recognize the different communication approaches necessary in different contexts and with different audiences. Planetary health partnerships will, by necessity, develop such translation skills within teams as members speak to their respective goals and values, but this skill is ultimately also necessary to promote stakeholder buy-in, access funding, and effect sustainable community and global change.

### Making partnerships work: Practical lessons from the Science of Team Science

Adopting a partnership approach in interdisciplinary teams can be challenging as members navigate goal setting, conflict resolution, and communication across disciplines, especially if those disciplines have a history of following the domination paradigm. A growing body of literature describes characteristics of particularly effective and innovative teams. This *Science of Team Science* [SciTS] was reviewed during its early emergence in a supplement to the *American Journal of Preventive Medicine* (Stokols, Hall, et al., 2008). SciTS is a valuable resource for interdisciplinary teams, and is relevant for adopting a partnership approach in these collaborations. For example, SciTS has pointed out the importance of “community coalitions” of researchers, practitioners, and community leaders collaborating on public health projects (Stokols, Misra, Moser, Hall, & Taylor, 2008, p. S104). The SciTS framing of these coalitions supports the importance of partnership approaches for complex health problems in which work must extend outside of academic silos to excite community action. The SciTS literature identifies factors that facilitate the effectiveness of interdisciplinary teams (Stokols, Misra, et al., 2008), and are particularly pertinent to interdisciplinary partnerships tackling complex challenges of planetary health.

SciTS describes engagement within teams, in which disciplines work together on a spectrum from independently combining projects (“multidisdplinarity”), to working jointly (“interdisciplinarity”), to synthesizing expertise to advance into new scientific territory (“transdisciplinarity”) (Stokols, Hall, et al., 2008, p. S78–79). Partnership approaches may have the greatest impact on complex planetary health problems by adopting truly transdisciplinary strategies, rather than the “parallel play” of multidisdplinarity. Further, the SciTS points out that these transdisciplinary teams should have a “shared vision and collective goals” (Stokols, Misra, et al., 2008, p. S109), which is particularly important for partnership approaches in which all team members must share in innovative idea generation.

Defining measurable, attainable, well-communicated goals is vital to effective partnership. Different disciplines or institutions may have their own respective goals within a team, but partners must ensure that these projects and goals are complementary. Realistically, research funding can make this kind of integrative goalsetting a challenge, as funding agencies may apply a “top-down” value system more in line with a domination approach. However, grants specifically seeking out interdisciplinary teams are beginning to appear (e.g. National Science Foundation Ecology and Evolution of Infectious Diseases), and may help foster transdisciplinary goal-setting. Regardless of funding source, partnerships can maximize their effectiveness by prioritizing shared goal-setting.

Team leaders are important for crafting project goals and vision, and leadership characteristics can have a major influence on the effectiveness of teams (Gray, 2008). In the context of the partnership paradigm, in which leaders must exercise “power with” instead of “power over” leadership styles, the characteristics of effective leadership are all the more pertinent. Leaders in effective partnerships should have an empowering, inclusive leadership style and skill in conflict resolution (Stokols, Misra, et al., 2008). Interpersonal conflict is an inherent challenge in any interdisciplinary team and must be managed carefully and conscientiously to maintain egalitarian partnerships. Addressing complex planetary health problems requires a range of expertise, and it is not expected that teams addressing these issues will have one permanent or static leader. Dynamic leadership may also be appropriate, with authority shifted or shared depending on the particular task (Stokols, Misra, et al., 2008). This must occur without causing a leadership vacuum, which can result in confusion over goals, tasks, priorities, and accountability. Partnership approaches promote effectiveness in complex problem solving by integrating expertise across disciplines, thereby making the empowering, transformational leadership style described by the SciTS literature a clear asset to these teams.

In the context of planetary health challenges, teams will be spread not only across different institutions, but different countries as well. For team leaders, this may mean having separate leadership hubs at each institution which can foster connections and facilitate communication. Teams can even use tools from social network analysis to evaluate these hub or broker roles (Gray, 2008). Particularly for planetary health partnerships, identifying intersectoral leaders or “champions” can also be important for coordinating across institutions, stakeholders, and/or community groups (Stokols, Misra, et al., 2008, p. S104).

The level of coordination required to effectively manage these complex, dispersed teams, however, requires technological support to facilitate clear, easy communication. Remote communication can create interpersonal challenges because of loss of social cues; these challenges may be magnified when communicating across professions, disciplines, languages, and cultures. To mitigate these challenges and promote partner trust and team identity, the SciTS literature suggests early in-person team interactions when partnerships are first forming, with periodic face-to-face interactions thereafter (Stokols, Misra, et al., 2008). Challenges in communication may also lead to higher coordination costs (Lee, Walsh, & Wang, 2015). Leaders of burgeoning partnerships should therefore evaluate the technology-readiness of participating team members and institutions early on to ensure clear, consistent communication.

Even ideal communication, however, cannot create effective partnerships if team members are resistant to collaboration. Thus, team collaboration readiness is also vital for interdisciplinary partnership success. Particularly in partnership approaches, in which all team members share in innovation and process, team members must acknowledge that each discipline - and team member - is equally valued and respected (Stokols, Misra, et al., 2008).

Defining roles for team members can be helpful for coordinating tasks and encouraging appreciation for the work and skills of others, but teams should use caution in creating overly specialized positions. Such specialization may reduce the ability of team members to integrate information across fields, limiting the truly transdisciplinary nature of partnership outcomes (Lee et al., 2015). Ultimately, by emphasizing the importance of shared goal development, empowering leadership, communication, and collaboration readiness, SciTS can help interdisciplinary teams adopt the partnership approaches necessary to address today’s planetary health challenges.

## CLIMATE CHANGE AND HEALTH CURRICULUM: A PARTNERSHIP-BASED PLANETARY HEALTH EXEMPLAR

Unless one comes from a culture that strongly orients toward partnership, it can be difficult to imagine how partnership-based planetary health solutions evolve. The following example may provide some insights.

Several faculty members in the Academic Health Center (AHC) of the University of Minnesota decided to focus their efforts on educating future health providers on the health impacts of climate change. This action arose from their deep conviction that climate change is one of the most critical planetary and public health crises facing humanity today.

A classic domination approach would have involved heavy-handed top-down communication and rigid models of leadership, but this method would not promote buy-in or shared responsibility. Instead, a partnership approach was used.

The project began with faculty considering the concept of “empathy for the user.” What type of world will future health professionals occupy? What will they need to know about climate change and planetary health to practice to their full potential? Instead of guessing student needs, faculty surveyed the students directly, asking, “How many of your current courses include climate change content? Do you believe climate change content should be added to health professional curriculum?” The response from students was overwhelming; they felt they need climate change content to prepare them to meet the needs of their future patients, and they were not being adequately prepared.

Armed with student data, the faculty brought their request to add climate change content to the curriculum of all the health professional schools across the Academic Health Center (AHC) to the Associate Deans. The vote to accept this proposal was unanimous.

The next step of the partnership approach involved selecting Climate Champions for each health profession school. The roles and responsibilities of the Climate Champions included:

- Surveying the current curriculum of their school to determine the degree to which climate change and health content is present.
- Determining the best placement for content and identifying faculty willing to teach the content.
- Working with other Climate Champions to co-create the climate change and health curriculum.

The identified Climate Champions formed interprofessional teams to work on the curriculum content. Eleven Climate Champions volunteered to work on slide decks corresponding to the major health impacts of climate change identified by the Centers for Disease Control and Prevention (2014). Topics include:

- Introduction and Resources
- Air Pollution
- Severe Weather
- Extreme Heat
- Increasing Allergens
- Changes in Vector Ecology
- Water Quality Impact
- Environmental Degradation
- Water and Food Supply

The slides were designed to be easy to use even if the faculty that taught the content had little knowledge of climate science. The topic is limited to 4–5 slides to alleviate concern that there is not enough time to cover the material. For many Climate Champions, this project was the first time they had worked with other health profession faculty to co-create a curriculum to be used across the AHC.

Dissemination of the completed curriculum also followed principles of partnership. It was made clear that this project was the result of Climate Champion collaboration and therefore one individual school could not take sole credit. In addition, the slides are stored on the Center for Global Health and Social Responsibility website (n.d.), a neutral AHC site which ensures that one school does not dominate.

The Climate Change and Health: An Interprofessional Response slide decks are open access. They are designed to be used by any individual or school in any location, local or global. The goal is to promote planetary health and contribute to climate change adaptation by creating easy-to-use resources available to all.

## CONCLUSION

Knowledge generation and dissemination in academic settings have traditionally been constrained by rigidly ranked discipline boundaries. Emerging planetary health crises have illuminated the limitations of this approach and the consequent gaps in finding effective solutions.

Cultural transformation theory (Eisler, 1988) offers academics and students a philosophical framework to move from disciplinary thinking to interdisciplinary partnerships. Research and teaching that orient toward partnership create opportunities for new insights and application of knowledge from one field to another. Furthermore, deep valuing of diverse expertise and mutual respect are the foundation for finding significant solutions to our most challenging planetary health problems.
